# Development and content validation of a self-completed, electronic Pediatric Asthma Symptom Diary

**DOI:** 10.1186/s41687-022-00432-3

**Published:** 2022-03-20

**Authors:** Marci Clark, Carla Romano, Oyebimpe Olayinka-Amao, Diane Whalley, Rebecca Crawford, Purnima Pathak, Caterina Brindicci, Kristin Garg, Kattayoun Kordy, Francois Everhard, Francesco Patalano, Zach Roesler, Thomas Sutton, Oskar Göransson, Ross Landles, Christel Naujoks, Jessica Marvel, Dorothy L. Keininger

**Affiliations:** 1 RTI Health Solutions, Ann Arbor, MI USA; 2 RTI Health Solutions, Research Triangle Park, NC USA; 3 RTI Health Solutions, Manchester, UK; 4 Novartis Ireland Ltd., Dublin, Ireland; 5 Novartis Pharma AG, Basel, Switzerland; 6 Novartis Pharmaceuticals, East Hanover, NJ USA; 7 frog, Milan, Italy

**Keywords:** Content validity, Electronic Pediatric Asthma Symptom Diary (ePASD), Interview, Measurement, Patient-reported outcome, Self-report

## Abstract

**Background:**

Childhood asthma is an important unmet need. To date, patient-reported outcome measures (PROMs) for children with asthma have used a combination of caregiver or proxy-reported and self-reported measures. No comprehensive measure is available to assess the severity and impact of daytime and nighttime asthma symptoms and rescue medication use for self-completion by children aged 6–11 years. This study aimed to develop a novel, interactive, electronic Pediatric Asthma Symptom Diary (ePASD) measuring self-reported key symptom severity and proximal impacts of asthma in young children with varying reading ability and disease severity, consistent with US Food and Drug Administration (FDA) PRO guidance and the International Society for Health Economics and Outcomes Research (ISPOR) good research practices.

**Methods:**

A targeted literature review and clinician interviews were undertaken to characterize symptoms and impacts experienced by children with mild-to-severe asthma. Concept elicitation interviews (CEIs) were conducted with 44 children and their caregivers (30 US; 14 UK). Following item and digital application development, the ePASD was assessed for relevance, understanding, and interpretability through cognitive debriefing interviews (CDIs) with 21 US children. Face validity/translatability assessments were also performed.

**Results:**

Key measurement concepts included cough, wheeze, difficulty breathing, chest tightness/discomfort, nighttime awakening, and daytime activity limitations. Concept saturation was reached during CEIs for primary asthma-related daytime and nighttime symptoms and core impacts. Most CDI participants found the ePASD items clear, understandable, and comprehensive. Standardized training is anticipated to facilitate reliable child self-report.

**Conclusion:**

The ePASD, a novel PROM for children aged 6–11 years with asthma, uses an innovative multimedia approach and has been developed in accordance with FDA PRO guidance and ISPOR good research practices, directly capturing the child’s self-reported asthma symptoms, impacts on daily activities and nighttime awakening, and rescue medication use.

**Supplementary Information:**

The online version contains supplementary material available at 10.1186/s41687-022-00432-3.

## Introduction

Asthma is a serious disease affecting more than 330 million people across all age groups worldwide [1, 2]. Childhood prevalence is high; nearly one in nine children have asthma in the United States (US) [1, 3], which represents an important unmet need. Asthma symptoms, generally evaluated via a daily diary, are a common endpoint for assessment in clinical trials evaluating pediatric asthma treatments [4]. Core daytime and nighttime symptoms of asthma include cough, wheeze, difficulty breathing, and chest tightness, although descriptors for these symptoms vary based on age and culture [5].

Reliable and valid assessment of asthma symptom severity, impact on daily activities and nighttime awakening, and rescue medication use contribute to the overall assessment of a patient’s asthma control [5]. Patient self-report of these concepts is considered preferable to caregiver report due to lack of agreement between patient and caregiver reports [6–8]. The US Food and Drug Administration (FDA) guidance on patient-reported outcomes (PROs), Patient-Focused Drug Development (PFDD) guidance discussion documents, and the International Society for Health Economics and Outcomes Research (ISPOR) task force report on PRO good research practices for the assessment of children and adolescents [9–13] provide a robust framework for development of pediatric PRO measures (PROMs), including daily diaries. Additionally, electronic administration of PROMs allows for development of interactive formats. Although electronic administration of PROMs can include risk of privacy loss, greater financial costs, and the need for a minimum digital ability of the participants, these disadvantages are offset by the electronic format having higher response rates, higher data quality, faster completion times, and being preferred by participants who prefer it over the traditional pen-and-paper format [14].

The results of a previously conducted targeted literature review, including an evaluation of asthma-specific PROMs, indicated that no comprehensive and validated diary meeting current FDA guidance was available to assess the severity and impact of asthma symptoms for self-completion by children aged 6–11 years. At the time this research was initiated, similar measures in development included the Pediatric Asthma Diary-Child (PAD-C) and the Pediatric Asthma Diary-Observer (PAD-O) [15]. However, there were known challenges with these measures, including the lack of availability for public use. Another issue was the need to have two different measures to cover the range of children aged 6–11 years, which would increase the difficulty of summarizing and interpreting results. Most importantly, although the PAD-C allows for self-completion, the PAD-O relies on observers for outcome data. The reported discordance between self-report from children and proxy report from parents for multiple measures [16–19] suggests that self-report by children may more accurately capture pediatric patients' perspectives. The ISPOR good research practices report [11] notes that children as young as 5 years old may be able to complete PROMs with good internal consistency and reliability. A single measure is needed to facilitate the self-report of asthma symptoms and impacts for pediatric patients aged 6–11 years with varying reading ability and disease severity.

The objective of this research was to develop a novel, electronic Pediatric Asthma Symptom Diary (ePASD) in accordance with current regulatory guidance and PRO good research practices [9–13]. The ePASD, administered via an interactive multimedia application on a tablet, can facilitate the self-report of key symptoms and proximal impacts of asthma by young children aged 6–11 years with mild, moderate, and severe asthma. A psychometric evaluation study is currently in progress to provide further support for key measurement properties (i.e., reliability, validity, and responsiveness), scoring, and preliminary responder definitions. The anticipated context of use for the ePASD is in future pediatric asthma clinical trials that are evaluating new treatments for patients aged 6–11 years.

## Methods

A targeted literature review and semistructured interviews with three pediatric expert clinicians were completed. A purposeful sampling approach [20] was taken in alignment with recent FDA guidance [21] to ensure enrolled participants experienced the key study concept (i.e., children with mild-to-severe asthma). Concept saturation (i.e., the point at which no new aspects of asthma symptoms or impacts were reported during the interviews) was documented [22]. The target sample size of 44 child-caregiver dyads was anticipated to establish concept saturation [23, 24].

Concept elicitation interviews (CEIs) were conducted with pediatric patients and their caregivers to elicit key symptoms and core impacts of asthma on daily activities. After ePASD item development, the digital application prototype was developed and tested. The ePASD was then tested in cognitive debriefing interviews (CDIs) with children with asthma for relevance, understanding, and interpretability. Face validity assessment (FVA) and translatability assessment were also performed to facilitate future translation into other languages. The initial FVA for the ePASD was conducted prior to the first round of CDIs with the use of screenshots of the ePASD. Changes were incorporated into the translatability assessment following review of the FVA results. The RTI International Institutional Review Board approved participant qualitative interviews. Expert clinician interviews were deemed exempt.

### Literature review and expert clinician interviews

A targeted literature review was conducted to identify key symptoms and impacts experienced by patients aged 6–11 years with asthma from the patient and caregiver perspectives. English-language articles available in the PubMed database and published between January 1, 2007, and March 21, 2017, were identified for potential full-text review (Additional file 1: Table S-1). The references of exemplar articles were reviewed for other relevant articles for inclusion. Additionally, an Embase conference abstract search was conducted with a focus on meeting proceedings from 2015 to 2017 (Additional file 1: Table S-2).

In-depth, 60-min telephone interviews were conducted with three expert clinicians based in the US, United Kingdom (UK), and Spain who had significant expertise in treating pediatric asthma. Each semistructured interview was led by two experienced interviewers (MC, OOA). The clinicians provided input on the daytime and nighttime asthma symptoms and associated impacts of greatest importance for evaluation of potential asthma treatment effects in pediatric clinical trials. Clinician perceptions were also sought on the reliability of patient self-report by age for the target age range and observed variability in symptoms and impacts.

### Concept elicitation interviews

Eligible individuals (Additional file 1: Table S-3) were scheduled for in-person interviews at two research facilities in the US (Raleigh, North Carolina; Southfield, Michigan) and one research facility in the UK (Stockport). Each semistructured interview was led by two experienced interviewers (US = MC, OOA; UK = DW, RC). Caregivers provided written informed consent for themselves and their child; children also provided their assent. Thirty child-caregiver dyads participated in interviews in the US, and 14 child-caregiver dyads participated in interviews in the UK. Each interview lasted approximately 1 h and was conducted by two experienced interviewers. The semistructured interviews began with open-ended questions about daily asthma symptoms and related proximal impacts. Follow-up questions focused on deeper understanding of specific symptoms and impacts to ensure that key symptoms were fully explored with each participant. Finally, caregivers described any impacts that asthma had on their or their child’s daily activities. Each interview was audio recorded, transcribed, and de-identified. Constant comparative analysis [25] was used to identify and compare dominant trends in each interview across the results of other interviews to generate themes or patterns in the way participants described their experiences with asthma. A data code book was used by one coder in the US and one coder in the UK to standardize analyses of field notes and transcripts across sites.

### Developing and testing the ePASD

The ePASD items were drafted for testing using primary symptoms and proximal (i.e., most closely related to the signs/symptoms that define the disease) [26] impacts identified from the targeted literature reviews, expert clinician interviews, and patient and caregiver CEIs. To preserve patient-derived concepts and terminology while ensuring content validity and ease of response, items were required to comply with a set of predefined principles used to guide instrument development (Additional file 1: Table S-4).

Once the items were drafted, a prototype of the ePASD digital application on the selected platform and electronic device (a tablet) was developed and tested by frog (Milan, Italy) using an iterative design research process in which children were presented with a wide variety of types of characters as stimuli to understand their desires before being presented with both a human-like character and a non-humanoid creature character to gauge their reactions and feelings. The results informed the development of a new character that incorporates characteristics of both types of characters: a non-human creature with human features that aim to be neutral in both gender and culture. Importantly, the interactive character contributes significantly to the novelty of the tool and helps facilitate the child’s independent self-completion of the diary items, regardless of reading ability. Specifically, the character demonstrates the concept captured by each question (e.g., coughing, wheezing), while its speech is synchronized with the audio so that the character moves its lips while speaking out the question on the screen. In addition, the character speaks out the corresponding response options when the child taps on each option. The child can also tap on the character on the tablet screen, which will prompt it to re-read the question aloud. The child must also tap “continue” on each screen to move on to the next question. The speaking character allows children to better follow the experience from start to finish and keeps their attention focused on one specific part of the interface, thus preventing distractions. The use of an interactive character with synchronized audio and demonstrated concepts allows the ePASD to be understood by children with a range of reading abilities, cognitive abilities, and asthma severity.

Two rounds of CDIs were conducted with 21 pediatric patients aged 6–11 years with mild, moderate, or severe asthma in the US to optimize the instructions, question wording, and response options for the US English version of the ePASD administered on the tablet. Each semistructured interview was led by experienced interviewers (MC, BO, CR). Eligibility requirements (Additional file 1: Table S-3) and interview format were the same as previously described for the CEIs. Child participants were encouraged to think aloud and describe their thought processes as they responded to each draft item, and probing questions were asked to understand how children interpret and select an answer for each item in the questionnaire. The interviews also offered the opportunity to identify additional concepts from participants not already captured by the ePASD. The first round of interviews was analyzed to identify patterns in the way participants interpreted and responded to each item and to determine the relevancy of ePASD items. Based on these interview results, revisions to the draft questionnaire were made and a draft ePASD administration manual was developed to provide guidance on facilitating standardized training. We evaluated the revised ePASD in the second round of interviews, and the results further informed the final ePASD. Each interview also offered an opportunity to identify additional concepts from participants. An item-tracking matrix was developed to illustrate how the text of the instructions, items, and response options changed (if at all) following each round of interviews, along with a description of the character where applicable.

Consistent with best practices for PROM development [22], FVA and translatability assessment were conducted. The FVA was conducted prior to the first round of CDIs, and the results informed ePASD revisions. In parallel with the Round 1 CDIs, a team of linguists representing 20 languages were asked to identify components of the revised ePASD that would be difficult to translate or that appeared to be culturally specific. Changes based on the translatability assessment results were incorporated into the ePASD prior to the second round of CDIs.

## Results

### Literature review and expert clinician interviews

The key asthma symptoms and impacts identified for measurement from the targeted literature review and expert clinician interviews are summarized below and detailed in Table 1.

**Table 1:** Key asthma symptoms and impacts identified for measurement from the literature review and expert clinician interviews

Symptom or Impact	Literature review	EC interviews
Primary asthma symptoms (daytime and nighttime)		
Cough	✓	✓
Wheeze	✓	✓
Difficulty breathing	✓	✓
Chest tightness/discomfort	✓	
Proximal impacts		
Nighttime awakening	✓^a^	✓
Physical and social activity limitations (exercise, running, playing, sports)	✓	✓
Rescue medication use		✓
Distal impacts		
Fatigue and lack of concentration (as result of disturbed sleep due to nighttime symptoms)	✓	
Lateness/absenteeism from school	✓	✓
Lower productivity at school	✓	
Missed social events	✓	✓
Health-related quality of life	✓	

*EC* expert clinician

^a^Nighttime awakening due primarily to cough and difficulty breathing were identified in the literature; nighttime awakening due to difficulty breathing, cough, and wheezing were primary symptoms reported by patients and/or caregivers to the expert clinicians interviewed

**Fig. 1:**
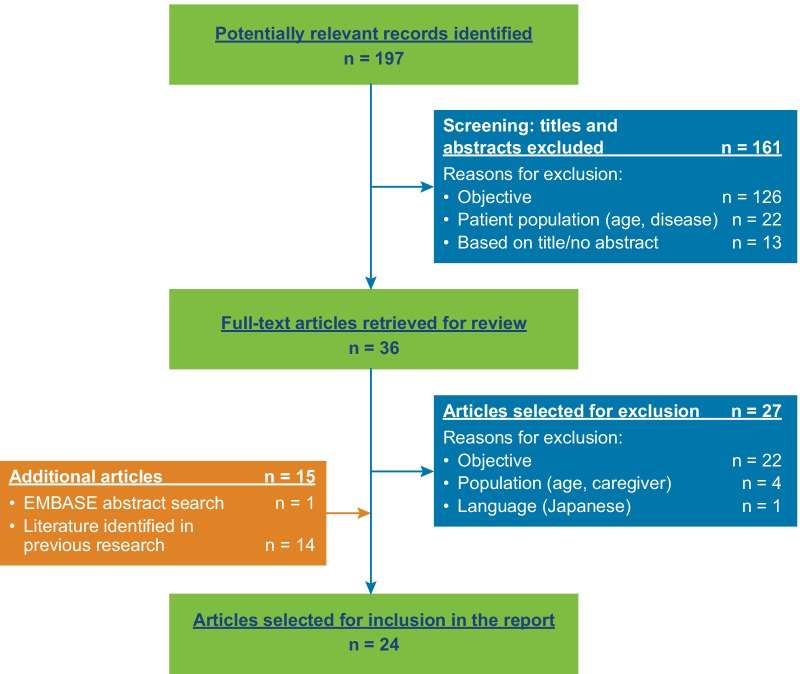
Flow diagram for included and excluded references

Twenty-four articles were reviewed and summarized (Fig. 1) [15, 27–49]. Most reported symptoms for patients aged 6–11 years with asthma were daytime and nighttime symptoms of cough, wheeze, difficulty breathing, and chest tightness/discomfort. Impacts included nighttime awakening and daytime activity limitations, fatigue and lack of concentration, lateness/absenteeism from school, lower productivity at school, missed social events, and diminished health-related quality of life (HRQOL). Key concepts recommended for assessment in the ePASD were cough, wheezing, difficulty breathing, and chest tightness/discomfort during the day and at night, as well as the impacts most proximal to the disease (i.e., nighttime awakening and activity limitations). Distal concepts, considered to be increasingly less related to the disease or effects of treatment [50] (e.g., fatigue and lack of concentration resulting from disturbed sleep, HRQOL), were not included due to the availability of existing questionnaires to capture these concepts.

The expert clinicians reported cough, wheeze, and difficulty breathing as the primary asthma symptoms most often described by young children with asthma and/or their caregivers. Primary asthma impacts included nighttime awakening or inability to sleep well due to nocturnal symptoms, physical and social activity limitations, missed school days, and the need to use rescue medication. Nighttime awakening due to primary asthma symptoms was especially considered to have a major impact on their lives. The clinicians generally endorsed the ability of pediatric patients to reliably self-report asthma symptoms, except in cases where patients are not awakened by nighttime symptoms. Additionally, all clinicians reported that reliability of patient self-report improves with age and agreed patient education meaningfully contributes to the ability of each patient to reliably self-report their symptoms and impacts regardless of age.

### Concept elicitation interviews with pediatric patients and their caregivers

Demographic and asthma severity information for the 44 child participants is presented in Table 2. The mean age of the total cohort was 8.2 years (range, 6–11 years), and most were male (68%). The sample included participants of different races and ethnicities, with a majority identifying as White (59%). Child participants’ asthma severity was well distributed from mild to severe, although slightly more participants had moderate asthma (43%). Concept saturation was reached for the primary asthma-related daytime (i.e., cough, difficulty breathing, wheezing, chest discomfort [i.e., chest pain and tightness], and activity limitations) and nighttime (i.e., cough, difficulty breathing, wheezing, and nighttime awakening) symptoms and proximal impacts reported by all child participants. Asthma symptoms and impacts observed and/or reported by all caregiver participants for their child with asthma were consistent with those reported by child participants.

**Table 2:** Concept elicitation interview child participant characteristics

Characteristics	US n = 30)	UK (n = 14)	Total (N = 44)
Age, years			
Mean	8.3	8.1	8.2
Range	6–11	6–11	6–11
Gender, n (%)			
Male	20 (67)	10 (71)	30 (68)
Female	10 (33)	4 (29)	14 (32)
Race/ethnicity, n (%)^a^			
Black	14 (47)	0 (0)	14 (32)
White	14 (47)	12 (86)	26 (59)
Hispanic	1 (3)	0 (0)	1 (2)
Asian	1 (3)	1 (7)	2 (5)
Mixed	0 (0)	1 (7)	1 (2)
Asthma severity, n (%)^b^			
Mild	9 (30)	5 (37)	14 (32)
Moderate	12 (40)	7 (50)	19 (43)
Severe	9 (30)	2 (14)	11 (25)
Education level: school grade (US)/Year (UK), n (%)			
1st/Year 2	6 (20)	3 (21)	9 (20)
2nd/Year 3	3 (10)	1 (7)	4 (9)
3rd/Year 4	4 (13)	5 (36)	9 (20)
4th/Year 5	8 (27)	1 (7)	9 (20)
5th/Year 6	3 (10)	0 (0.0)	3 (7)
6th/Year 7	5 (17)	4 (29)	9 (20)
9th/Year 10	1 (3)	0 (0.0)	1 (2)

Percentages total slightly less or greater than 100% due to rounding

*UK* United Kingdom, *US* United States

^a^Ethnicity and race were collected together and not differentiated

^b^Asthma severity levels defined based on Global Initiative for Asthma 2018 guidelines and child’s daily asthma controller medication use at screening as follows: Mild = low-dose inhaled corticosteroid (ICS) alone or low-dose ICS + leukotriene receptor antagonist (LTRA alone; Moderate = low-dose ICS/long-acting beta agonist (LABA), LTRA, medium-dose ICS alone or high-dose ICS alone; Severe = medium-dose ICS/LABA, medium-dose ICS + LTRA, high-dose ICS/LABA or high-dose ICS + LTRA

### Developing and testing the ePASD

**Fig. 2:**
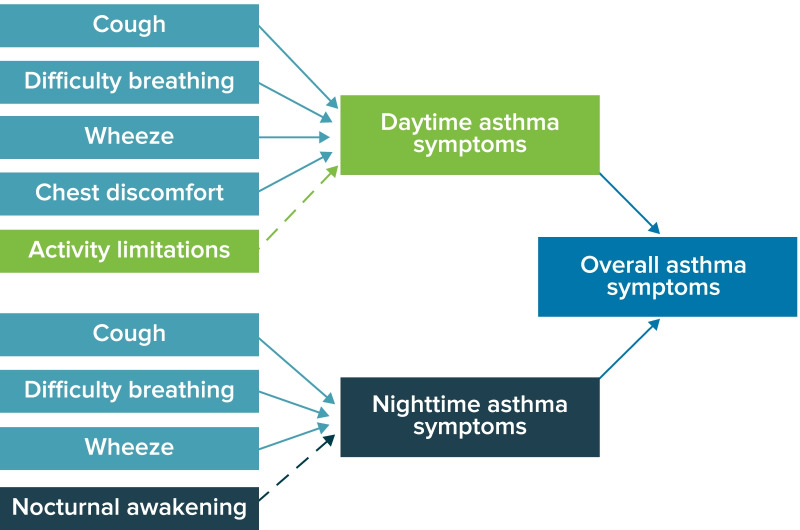
ePASD draft conceptual framework. *ePASD* electronic Pediatric Asthma Symptom Diary

Based on findings from the literature review, interviews with pediatric asthma clinical experts, and CEIs, a conceptual framework for the ePASD (Fig. 2) was developed. Items for inclusion in the ePASD were generated to reflect key symptoms and impacts experienced by children aged 6–11 years with mild to severe asthma. Specifically, the concepts identified for the draft ePASD item pool included those reported by the child interview participants as most frequently occurring and most bothersome. The draft daytime diary (completed each evening) included items assessing daytime asthma symptoms (cough, wheeze, chest pain/tightness, difficulty breathing) and activity limitations (Table 3). The draft nighttime diary (completed each morning) included items assessing nocturnal asthma symptoms (cough, wheeze, difficulty breathing) and nighttime awakening due to asthma (Table 4). An item assessing rescue medication use was also added to the daytime and nighttime diaries because of the importance of capturing this information directly from the patient and assessing overall asthma control. The items were developed in accordance with Global Initiative for Asthma (GINA) 2019 guidelines [51], FDA PRO and PFDD guidance documents [9, 10, 12, 13], and the ISPOR PRO good research practices task force report [11]. The simple face, text, and verbal response scale included varies by item. Minor editorial changes were incorporated based on FVA and translatability assessment.

**Table 3:** Draft ePASD daytime symptom and proximal activity items with selected quotes from child concept elicitation interview participants

Item	US quotes	UK quotes
*Daytime diary (daytime symptoms)*		
How was your cough today?	[Cough], it’s usually like louder. It’s like a regular cough but probably a little bit louder	Sometimes it’s like only the slightest cough and sometimes it’s like I keep coughing for ages and it starts to hurts
Did you wheeze today?	It’s basically like when I breathe but it’s like because sometimes when I breathe, it like stuff in my throat so it’s like a wheeze or whatever. It’s kind of like pretty low pitch	My breathing goes a bit like a kazoo
Did your chest hurt today?	And if I run too much, my chest is going to start hurting. My chest gets stuffy. It gets tight I cough, and I cough, and I cough, and I gasp, and I wheeze, and then my lungs hurt	I was coughing too hard, like this, and my chest started to hurt
How was your breathing today?	My breath was like much colder, it felt like, and my chest couldn’t inhale or exhale. Yeah, it was that bad… I think it’s more when I’m actually active	I just can’t breathe and I feel like something’s blocking my throat or something and I can’t get it out
How hard was running, playing, or doing sports today because of your asthma?	It’s normally when I’m playing, like, if I’m at the middle of a basketball game or something else. It just makes me stop running or something	When I’m running or playing sport. When you have to run dead fast or sometimes at night when I’m moving around a lot
Why didn’t you run, play, or do sports today?	It’s a little bit hard. And it’s been times I would want to do stuff and then I can’t because of my asthma. And it’s harder for me to handle it during gym	Or sometimes I will just take the whole PE session off because it [difficulty breathing] gets too bad

*ePASD* electronic Pediatric Asthma Symptom Diary, *PE* physical education, *UK* United Kingdom, *US* United States

**Table 4:** Draft ePASD nighttime symptom and proximal activity items with selected quotes from child concept elicitation interview participants

Item	US quotes	UK quotes
*Nighttime diary (nighttime symptoms)*		
How was your cough last night?	Sometimes I cough a lot at night	I just like wake up in the night because I’m coughing so much in my sleep
Did you wheeze last night?	If I try to breathe in or breathe out, I can hear it [nighttime wheezing] Well [wheezing], at nighttime, sometimes	In the middle of the night, um, I start wheezing when I’m breathing and I wake up and try to cough it out and I got really annoyed because, um, I got really annoyed because when I started coughing my chest started hurting
How was your breathing last night?	[Breathing too fast]. Probably at night or in the morning. This morning it didn’t happen, but last night	When my asthma is getting a bit wheezy I am struggling to breathe and it’s hard [coughing sound] because it’s hard to breathe, because my chest gets really bad and normally at night and I start to breathe really heavily
Did you wake up last night because of your asthma?	Sometimes in the night, I’ll wake up, like, in the middle of the night and start gasping and coughing, couldn’t breathe	I just like wake up in the night because I’m coughing so much in my sleep

*ePASD* electronic Pediatric Asthma Symptom Diary, *UK* United Kingdom, *US* United States

Demographic and asthma severity information for the 21 child CDI participants are presented in Table 5. The mean age of the total sample was 8.2 years (range, 6–11 years) and slightly more than half the participants were female (57%). The study population recruited was again diverse, with a majority of participants identifying as Black (57%). Participants’ asthma severity ranged from mild to severe, although slightly more participants had moderate asthma (43.2%).

**Table 5:** Cognitive debriefing child participants characteristics

Characteristics	Philadelphia, PA, US Round 1 (n = 8)	Southfield, MI, US Round 2^a^ (n = 13)	Total (N = 21)
Age, years			
Mean	8.5	8.0	8.2
Range	6–11	6–11	6–11
Sex, n (%)			
Male	4 (50)	5 (38)	9 (43)
Female	4 (50)	8 (62)	12 (57)
Race, n (%)			
Black	4 (50)	8 (62)	12 (57)
White	3 (38)	4 (31)	7 (33)
Other^b^	1 (13)	1 (8)	2 (10)
Hispanic ethnicity, n (%)^c^			
Yes	1 (13)	0 (0)	1 (5)
No	7 (88)	13 (100)	20 (95)
Asthma severity, n (%)^d^			
Mild	2 (25)	3 (23)	5 (24)
Moderate	2 (25)	7 (54)	9 (43)
Severe	4 (50)	3 (23)	7 (33)
Reading skill level, n (%)			
Can read alone	5 (63)	9 (69)	14 (67)
Can read but with some difficulty	2 (25)	2 (15)	4 (19)
Can only read a few words	1 (13)	2 (15)	3 (14)

*ePASD* electronic Pediatric Asthma Symptom Diary, *MI* Michigan, *PA* Pennsylvania, *US* United States

^a^Two of the 13 children (ages 6 [can read a few words] and 7 [can read but with difficulty]) participating in Round 2 interviews were unable to complete the cognitive interview for the ePASD nighttime diary; however, both of these children were able to successfully self-complete all of the ePASD questions on the tablet

^b^Round 1 participant that selected “Other” for race specified mixed race of White/Black; Round 2 participant that selected “Other” for race specified mixed race of White/Asian

^c^Ethnicity was collected separately from race

^d^Asthma severity levels defined based on Global Initiative for Asthma 2018 guidelines and child’s daily asthma controller medication use at screening as follows: Mild = low-dose inhaled corticosteroid (ICS) alone or leukotriene receptor antagonist (LTRA) alone; Moderate = low-dose ICS/ long-acting beta agonist (LABA), low-dose ICS + LTRA, medium-dose ICS alone or high-dose ICS alone; Severe = medium-dose ICS/LABA, medium-dose ICS + LTRA, high-dose ICS/LABA or high-dose ICS + LTRA

**Fig. 3:**
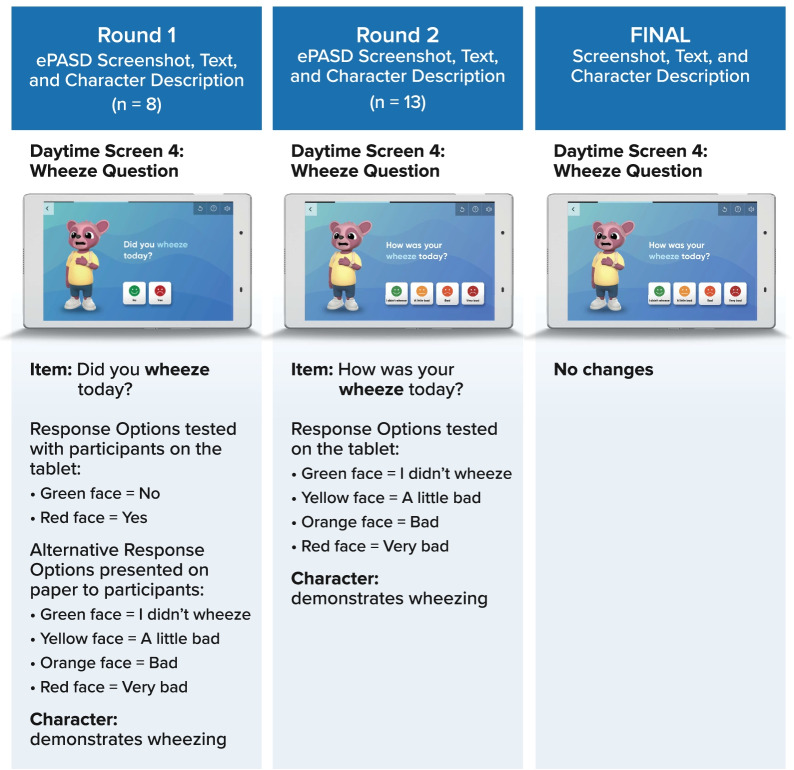
Depiction of ePASD wheeze daytime item through each round of cognitive debriefing interviews and the final item version. *ePASD* electronic Pediatric Asthma Symptom Diary. Image reproduced with permission from *Novartis Pharma AG*

All Round 1 (n = 8) and Round 2 (n = 13) participants completed the cognitive debriefing of the daytime ePASD items using the tablet. All Round 1 participants (n = 8) and the majority (n = 11) of Round 2 participants completed the cognitive debriefing of the nighttime ePASD items using the tablet. However, a 6-year-old and a 7-year-old participant in Round 2 experienced difficulty with the cognitive debriefing process. These two participants completed debriefing for only the daytime questionnaire. Most (n = 6) Round 1 participants easily understood and accurately interpreted the daytime instructions. All Round 1 participants easily understood and accurately interpreted the nighttime instructions, including the recall period “last night.” While the majority of CDI participants across both rounds reported that most of the ePASD items were relevant, clear, and easy to understand (see supportive quotes in Additional file 1: Tables S-5 and S-6), a few patients, ages 6–10 years, in both interview rounds had trouble recalling what “today” or “last night” meant when answering some of the questions as well as discerning their rescue inhaler from their regular daily inhaled corticosteroid when asked about rescue medication use. Additionally, five of the Round 1 participants had difficulty with the daytime and nighttime wheeze items (“Did you wheeze today/last night?”) and with the “yes” and “no” face/text/verbal scale options initially proposed, reporting that their wheeze had different levels of severity. These participants preferred the items and response options be changed to “How was your wheeze today/last night?” with four alternative face/text response options illustrated on paper (Fig. 3). The alternative items with revised scale were implemented on the tablet for Round 2 testing and retained for the final ePASD based on participant feedback.

Overall, across both rounds of interviews, the participants were receptive to using an electronic tablet to complete the ePASD daytime and nighttime items, and no important asthma symptoms or impacts were noted as missing. Participant input from each round was used to optimize the instructions, question wording, and response options for the US English version of the ePASD (Fig. 3). Furthermore, child participants were receptive to using the tablet to complete the ePASD and generally reported that the animated character that reads the instructions, questions, and response options aloud and demonstrated each asthma symptom, proximal impact, and use of the rescue inhaler was helpful.

The final version of the ePASD included seven items in the morning diary and five items in the nighttime diary. All items in the ePASD were designed to be self-completed by children with a range of different reading abilities without help from caregivers. The administration manual developed to train children and their caregivers on the ePASD is anticipated to further standardize patient education on both instructional and disease terminology used in the ePASD and further facilitate self-completion of the measure.

## Discussion

The ePASD is a novel PROM designed to facilitate self-completion by children as young as 6 years of age with mild, moderate, or severe asthma who may or may not read independently using an interactive electronic application on a tablet. The development of this unique, multimedia instrument was supported by a robust process that included a targeted literature review, semistructured interviews with expert clinicians, CEIs with pediatric patients and their caregivers to elicit key symptoms and impacts, development of the electronic application (including the interactive character [data on file]), and CDIs with pediatric patients to develop and test the ePASD items. The ePASD has been developed in accordance with FDA guidance [9, 10, 12, 13] and the ISPOR pediatric PRO good research practices task force report [11].

There have been several pediatric asthma diaries developed for children aged 6–11 years, including the child-completed Pediatric Asthma Diary (PAD) (aged 6–14 years), the child-completed PAD-C (aged 8–11), and parent/caregiver completed PAD-O (aged 4–11 years) [15, 52]. Although the PAD allows for self-report by children as young as 6 years, this older measure was not developed using recent FDA guidance and was not capable of detecting differences in nighttime symptoms between stable patients and unstable patients who required additional medication during validation assessment [52]. The ePASD incorporates similar key symptoms and impacts of asthma as the PAD-C and PAD-O; however, the PAD-O prohibits self-report and the PAD-C has a lower age limit of 8 years [15]. A key feature of the ePASD is that the patient experience of asthma symptoms and impacts is captured directly from children as young as 6 years without influence from caregivers acting as proxies. Parents/caregivers may perceive and report asthma-related activity limitations differently than their child [53], which bolsters the importance of self-report by children. Additionally, child self-report facilitates capture of the most meaningful treatment benefits that can only be experienced and described by the patient.

The CDIs demonstrated the ePASD to be appropriate and feasible for children aged 6–11 years with a range of reading abilities and provided evidence of the content validity of the measure. All instructions, questions, and answer options are read aloud to the child by the interactive character in the application and can be replayed by the child on the device as many times as needed to facilitate understanding and self-completion of all ePASD questions. Additionally, the character demonstration of each asthma symptom (i.e., cough, wheeze, difficulty breathing, chest discomfort) and impact (nighttime awakening and daytime activity limitation due to asthma) standardizes patient comprehension of the text and further facilitates self-report. The visual and audio demonstration of symptoms is especially important for the youngest patients with asthma who cannot read or who have difficulty reading independently. The majority of CDI participants found the ePASD items clear, understandable, and comprehensive. Although two of the youngest participants (a 6-year-old and a 7-year-old) in the second round of interviews had difficulty with the cognitive debriefing exercise, and only provided feedback on the ePASD daytime diary items, these participants were able to successfully complete the ePASD nighttime diary items on their own, which offered support for the ability of even the youngest children to self-complete this questionnaire. The difficulty with the cognitive debriefing exercise reflected challenges with the think-aloud cognitive debriefing component and not with self-completion of the ePASD.

The CDI results supported development of an ePASD administration manual to provide standardized training for a child’s self-administration of the ePASD for future clinical study site coordinators, caregivers, and children with asthma. The manual and training would facilitate a standardized data collection approach that could reduce potential variability across all future study participants and clinic sites, as well as improve overall data quality, reliability, and validity. Key components of the administration manual include standardized definitions for the two ePASD recall periods (i.e., “today” and “last night”) as well as key terms (i.e., asthma, wheeze, chest discomfort, and rescue inhaler). Furthermore, instructions are included for the study coordinator to identify the designated rescue inhaler with a sticker so that the child knows which inhaler to think about when answering questions about rescue medication use.

A limitation of this study is that the development of the ePASD was primarily focused on English-speaking US and UK participants; however, the favorable results of the translatability assessment provide confidence in future translations. A prospective, longitudinal, psychometric evaluation study is currently in progress to provide further support for key measurement properties (i.e., reliability, validity, and responsiveness) that are consistent with FDA PRO guidance [9]. Information about the structure, scoring, performance, and interpretation of the ePASD, including preliminary responder definitions, will be established in the psychometric study and will provide the groundwork needed for ePASD implementation in the context of future clinical trials for pediatric asthma treatment. Additionally, translation and linguistic validation of the ePASD consistent with current standards [54] is planned for global clinical trial use.

## Conclusion

The development and testing of the ePASD generated evidence supporting the appropriateness and feasibility of this measure for children aged 6–11 years with mild, moderate, or severe asthma to self-report regardless of their ability to read independently. This measure is the first novel, multimedia PROM developed in accordance with FDA PRO guidance and good research practices to directly capture a child’s asthma symptoms, impacts, and rescue medication use in an engaging and interactive manner.

## Supplementary Information

**Additional file 1:** Supplementary Material.

## Data Availability

Data are primarily in the form of transcripts and cannot be made available in order to protect participant privacy in accordance with the principles of the Belmont Report.
