# Supplementary material for: Development and content validation of a self-completed, electronic Pediatric Asthma Symptom Diary
Source: J Patient Rep Outcomes. 2022 Mar 20;6:25. doi: 10.1186/s41687-022-00432-3 (PMC8934788; doi:10.1186/s41687-022-00432-3)
Supplement: Supplementary file 1 — Additional file 1: Supplementary Material. [file 41687_2022_432_MOESM1_ESM.docx]

# SUPPLEMENTARY MATERIAL

Table S-1. PubMed Literature Search Strategy: Symptoms and Impacts

| **Search** | **Terms** | **Records** |
| --- | --- | --- |
| **Disease** | |  |
|  | #1 “Asthma”[Majr] OR asthma[Title] Publication date from 2007/01/01 | **30,829** |
| **Population** | |  |
|  | #2 #1 AND (“Child”[Mesh] OR child*[Title] OR pediatric[Title] OR paediatric[Title]) Publication date from 2007/01/01 | **9,883** |
| **Symptoms** | |  |
|  | #3 #2 AND ((“Quality of Life”[Mesh] OR “quality of life”[Text Word] OR “life quality”[Text Word] OR “QOL”[Text Word] OR “HRQOL”[Text Word]) AND (“Signs and Symptoms”[Mesh] OR symptom*[Title] OR “PRO”[Text Word] OR “patient reported outcome”[Text Word] OR symptom impact*[Text Word] OR “impact of symptoms”[Text Word] OR “impacts of symptoms”[Text Word] OR “consequences of symptoms”[Text Word] OR “consequence of symptoms”[Text Word] OR “symptom consequences”[Text Word]) Publication date from 2007/01/01 | **69** |
| **Study types** | |  |
|  | #4 #3 AND (“Observational Studies as Topic”[Majr] OR “Qualitative Research”[Majr] OR “Self Report”[Majr] OR “Focus Groups”[Majr] OR “Interviews as Topic”[Majr] OR “Surveys and Questionnaires”[Majr] OR “qualitative research”[Text Word] OR qualitative stud*[Text Word] OR diary[Text Word] OR diaries[Text Word] OR questionnaire*[Title] OR survey*[Title] OR self report*[Text Word] OR patient report*[Text Word] OR proxy report*[Text Word] OR parent report*[Text Word] OR caregiver report*[Text Word] OR observer report*[Text Word] OR focus group*[Text Word] OR interview*[Title] OR observational stud*[Text Word] OR “Observational Study”[Publication Type] OR “Interview”[Publication Type]) Publication date from 2007/01/01 | **20** |
| **Guidelines** |  |  |
|  | #5 #2 AND (“Guidelines as Topic”[Mesh] OR practice guideline*[Text Word] OR clinical guideline*[Text Word] OR guideline*[Title] OR “Practice Guideline”[Publication Type]) Publication date from 2012/01/01 | **170** |
| **Exclusions** | |  |
|  | #6 “Animals”[Mesh] NOT “Humans”[Mesh] Publication date from 2007/01/01  #7 “Comment”[Publication Type] OR “Letter”[Publication Type] Publication date from 2007/01/01 | **1,049,794**  **518,110** |
| **Total** | |  |
|  | #8 ((#4 OR #5) NOT (#6 OR #7)) | **197** |

Table S-2. Conference Proceedings Search Strategy

| **Search** | **Terms** | **Records** |
| --- | --- | --- |
| **Disease** | |  |
|  | #1 ‘asthma’/exp/mj OR asthma:ti AND [english]/lim AND [embase]/lim AND [2007-2017]/py | **56,007** |
| **Population** | |  |
|  | #2 #1 AND (‘child’/exp OR child*:ti OR pediatric:ti OR paediatric:ti) AND [english]/lim AND [embase]/lim AND [2007-2017]/py | **16,562** |
| **Symptoms** | |  |
|  | #3 #2 AND (‘quality of life’/exp OR ‘quality of life’:de,ab,ti OR ‘life quality’:de,ab,ti OR ‘qol’:de,ab,ti OR ‘hrqol’:de,ab,ti) AND (‘physical disease by body function’/exp OR symptom*:ti OR ‘pro’:de,ab,ti OR ‘patient reported outcome’:de,ab,ti OR (symptom NEXT/1 impact*):de,ab,ti OR ‘impact of symptoms’:de,ab,ti OR ‘impacts of symptoms’:de,ab,ti OR ‘consequences of symptoms’:de,ab,ti OR ‘consequence of symptoms’:de,ab,ti OR ‘symptom consequences’:de,ab,ti) AND [english]/lim AND [embase]/lim AND [2007-2017]/py | **1,115** |
| **Study types** | |  |
|  | #4 #3 AND (‘observational study’/exp/mj OR ‘qualitative research’/exp/mj OR ‘self report’/exp/mj OR ‘information processing’/exp/mj OR ‘interview’/exp/mj OR ‘questionnaire’/exp/mj OR ‘qualitative research’:de,ab,ti OR (qualitative NEXT/1 stud*):de,ab,ti OR diary:de,ab,ti OR diaries:de,ab,ti OR questionnaire*:ti OR survey*:ti OR (self NEXT/1 report*):de,ab,ti OR (patient NEXT/1 report*):de,ab,ti OR (proxy NEXT/1 report*):de,ab,ti OR (parent NEXT/1 report*):de,ab,ti OR (caregiver NEXT/1 report*):de,ab,ti OR (observer NEXT/1 report*):de,ab,ti OR (focus NEXT/1 group*):de,ab,ti OR interview*:ti OR (observational NEXT/1 stud*):de,ab,ti) AND [english]/lim AND [embase]/lim AND [2007-2017]/py | **260** |
| **Guidelines** | |  |
|  | #5 #2 AND (‘practice guideline’/exp OR (practice NEXT/1 guideline*):de,ab,ti OR (clinical NEXT/1 guideline*):de,ab,ti OR guideline*:ti) AND [english]/lim AND [embase]/lim AND [2012-2017]/py | **388** |
| **Exclusions** | |  |
|  | #6 ‘animal’/exp NOT ‘human’/exp AND [english]/lim AND [embase]/lim AND [2007-2017]/py  #7 comment*:ti OR letter:it AND [english]/lim AND [embase]/lim AND [2007-2017]/py | **1,129,057**  **384,760** |
| **Total** | |  |
|  | #8 ((#4 OR #5) NOT (#6 OR #7))  #9 #8 AND (‘conference paper’:it OR ‘conference abstract’:it) AND [2015-2017]/py  #10 #9 AND (‘2015 annual meeting of the american academy of allergy, asthma and immunology, aaaai 2015’:nc OR ‘2016 annual meeting of the american academy of allergy, asthma and immunology, aaaai 2016’:nc OR ‘american thoracic society international conference, ats 2015’:nc OR ‘european respiratory society annual congress 2015’:nc) | **629**  **62**  **23** |

Table S-3. Child Participant Inclusion and Exclusion Criteria^a^

| Inclusion criteria |
| --- |
| - 6-11 years of age |
| - Mild, moderate, or severe persistent asthma^b^ that was diagnosed by a physician ≥ 6 months ago |
| - During the past 4 weeks, have experienced ≥ 1 of the following: |
| - daytime asthma symptoms more than twice per week - night waking because of asthma - use of rescue medication more than twice per week - any activity limitation due to asthma |
| - Had been taking at least a low dose of inhaled corticosteroid or a leukotriene receptor for ≥ 3 months - Able to read, understand and provide responses in English - Have an adult primary caregiver who can read, understand, and provide responses in English and is willing to participate in the interview with his/her child |
| Exclusion criteria |
| - History of chronic pulmonary disease other than asthma or another condition that affects lung function |
| - Not able to be compliant with the study procedures |
| - Had any medical or mental disorder, situation, or diagnosis that could have interfered with the proper completion of the qualitative interview |

^a^ Based on caregiver report.

^b^ Asthma severity classification was based on the child’s asthma controller medication and assigned concurrent with Global Initiative for Asthma (GINA) 2018 guidelines: GINA report, global strategy for asthma management and prevention. Retrieved November 5, 2020, from <https://ginasthma.org/wp-content/uploads/2019/01/2018-GINA.pdf>

Table S-4. Principles for Instrument Development

| Principles used to guide development of the ePASD |
| --- |
| - Content must be: - relevant to the assessment of primary asthma symptoms and proximal impacts only - applicable to target audience (i.e., across age, gender, socioeconomic status, and educational level) |
| - Succinct, concise, and efficient wording |
| - Item structure and content should cover the breadth of asthma symptom severity |
| - Questions should be logically linked to the response options |
| - Concepts in the item should be pertinent (and equally understandable) to patients across the spectrum of the disease |
| - Culturally generic |
| - Generalizable across population |
| - Pertinent to potential effects of treatment |
| - Accurate over recall period |
| - Underlying concept has potential to change over time |
| - Independent of other constructs |
| - Minimal cognitive load |
| - Appropriate ordering |

ePASD = electronic Pediatric Asthma Symptom Diary.

Table S-5. Draft ePASD Daytime Symptom and Proximal Activity Items With Supportive Quotes From Child Cognitive Debriefing Interview Participants

| Item | Supportive Quotes |
| --- | --- |
| **Daytime diary (daytime symptoms)** |  |
| How was your cough today? | - *How would I rate my coughing today? [Round 1, IDI #7]* - *How was my cough today. [Round 2, IDI #11]* |
| How was your wheeze today?^a^ | - *It’s asking how was my wheezing today. [Round 2, IDI #01]* - *It was asking me how much I wheezed today. [Round 2, IDI #04]* |
| Did your chest hurt today? | - *Did your chest hurt at all with wheezing and stuff and coughing. [Round 1, IDI #5]* - *It’s asking me if I had tightness, discomfort, or chest pain in my chest. [Round 2, IDI #4]* |
| How was your breathing today? | - *It’s asking how I was breathing today. [Round 1, IDI #3]* - *It’s asking me, like, what was the rate of my breathing and how it felt today. [Round 2, IDI #1]* |
| How hard was running, playing, or doing sports today because of your asthma? | - *If it was hard to do any physical activities because of my asthma. [Round 1, IDI #7]* - *It’s asking me how hard it was or how annoying it was to do sports because of my asthma, like if I had to stop a lot and take breathing treatments. [Round 2, IDI #4]* |
| Why didn’t you run, play, or do sports today? | - *It wants to know why I didn’t do any sports, playing, or running today [Round 1, IDI#1]* - *It's why didn't I do any today. [Round 1, IDI#7]* |

ePASD = electronic Pediatric Asthma Symptom Diary; IDI = in-depth interview.

^a^ This version of the item was tested only in Round 2 and was retained for the final ePASD.

Table S-6. Draft ePASD Nighttime Symptom and Proximal Activity Items With Supportive Quotes From Child Cognitive Debriefing Interview Participants

| Item | Supportive Quotes |
| --- | --- |
| **Nighttime diary (nighttime symptoms)** |  |
| How was your cough last night? | - *If I coughed last night. I didn’t’ cough. [Round 1, IDI #07]* - *It’s asking me how my cough was last night. [Round 2, IDI #01]* |
| How was your wheeze last night?^a^ | - *How was I…how was my wheezing last night. [Round 2, IDI#02]* |
| How was your breathing last night? | - *It’s asking how well did you breathe last night. [Round 1, IDI #05]* - *It’s asking me if my breathing was hard or easy. [Round 2, IDI #04]* |
| Did you wake up last night because of your asthma? | - *It’s asking me whether or not I woke up last night because of my asthma. [Round 2, IDI #04]* |

ePASD = electronic Pediatric Asthma Symptom Diary; IDI = in-depth interview.

^a^ This version of the item was tested only in Round 2 and was retained for the final ePASD.
